# fENko-Kae01 is a flagellum-specific jumbo phage infecting *Klebsiella aerogenes*

**DOI:** 10.1186/s12866-024-03387-1

**Published:** 2024-07-01

**Authors:** Kira Ranta, Mikael Skurnik, Saija Kiljunen

**Affiliations:** 1 HUS Diagnostic Center, Clinical Microbiology, University of Helsinki and Helsinki University Hospital, Helsinki, Finland; 2 Human Microbiome Research Program, Research Program Unit, Faculty of Medicine, University of Helsinki, Helsinki, Finland

**Keywords:** Bacteriophage, Jumbo phage, *Klebsiella aerogenes*, Flagellum-dependent, Phage-binding receptor, Phage resistance, Phage therapy

## Abstract

**Background:**

*Klebsiella aerogenes* is an opportunistic pathogen that causes a wide variety of infections. Due to the rising problem of antibiotic resistance, novel antibiotics and strategies to combat bacterial infections are needed. Host-specific bacteriophages are natural enemies of bacteria and can be used in phage therapy as an alternative form of treatment against bacterial infections. Jumbo phages are defined as phages with genomes larger than 200 kb. Relatively few studies have been done on jumbo phages compared to smaller phages.

**Results:**

A novel phage, fENko-Kae01, was isolated from a commercial phage cocktail. Genomic analysis revealed that fENko-Kae01 is a lytic jumbo phage with a 360 kb genome encoding 578 predicted genes. No highly similar phage genomes were identified and fENko-Kae01 may be a completely new genus representative. No known genes associated with lysogenic life cycle, bacterial virulence, or antibiotic resistance were identified. The phage had myovirus morphology and a narrow host range. Phage resistant bacterial mutants emerged under phage selection. Whole genome sequencing revealed that the biogenesis of the flagellum was affected in four mutants and the lack of functional flagellum was confirmed in motility assays. Furthermore, phage fENKo-Kae01 failed to adsorb on the non-motile mutants indicating that the bacterial flagellum is the phage-binding receptor.

**Conclusions:**

fENko-Kae01 is a novel jumbo bacteriophage that is considered safe for phage therapy. fENko-Kae01 uses the flagellum as the phage-binding receptor and may represent a completely novel genus.

**Supplementary Information:**

The online version contains supplementary material available at 10.1186/s12866-024-03387-1.

## Introduction

*Klebsiella aerogenes* (formerly *Enterobacter aerogenes*) [1] belongs to the gram-negative family Enterobacteriaceae. *K. aerogenes* is an opportunistic pathogen that causes a wide variety of infections, including pneumonia, bacteremia, urinary tract infections and surgical site infections. Patients who are immunosuppressed or stay at the hospital for a prolonged time are at special risk of developing a *K. aerogenes* infection [2–4].

The most common cause of multidrug-resistant (MDR) bacterial infections are members of the ESKAPE pathogen group [5, 6]. Traditionally, the ESKAPE pathogen group includes *Enterococcus faecium, Staphylococcus aureus, Klebsiella pneumoniae, Acinetobacter baumannii, Pseudomonas aeruginosa* and *Enterobacter* spp [7, 8]. Nowadays, however, the whole order Enterobacterales is often included to the ESKAPE pathogens [6, 9]. Due to the rising problem of antibiotic resistance, novel antibiotics and strategies to combat bacterial infections are needed. In 2017 the World Health Organization (WHO) published a list of bacteria for which new antibiotics are urgently needed [10]. MDR Enterobacteriaceae are listed as priority 1 and are in most need of novel antibiotics.

Bacteriophages (phages) are natural enemies of bacteria and can be used in phage therapy as an alternative treatment against bacterial infections, especially those caused by MDR bacteria [11, 12]. Briefly, phages bind to a receptor on the bacterial surface, eject their genome into the bacterial cell, and replicate within the bacterium, after which new virus particles are released via cell lysis. Phages can target any structural element on the bacterial cell surface. Phages are host specific and the ability to bind to a single or multiple receptors correlates with how narrow or broad the host range of a specific phage is [13, 14].

Flagellotropic phages attach to the bacterial flagellum to initiate infection. The phage usually attaches to the distal part of the flagellum and, through the rotation of the flagellum, moves along the shaft of the flagellum towards the cell surface. Flagellotropic phages have diverse mechanisms of attachment to the flagellum. When reaching the bacterial cell surface, the phage then supposedly binds to a secondary receptor to continue infection. The secondary receptors of flagellotropic phages are poorly known [15, 16], only recently the lipooligosaccharide of *Campylobacter jejuni* was identified as the secondary receptor of phage F341 [17].

As phages are natural enemies of bacteria, bacteria are able to develop resistance against phages to ensure their own survival. Phage resistant bacteria usually undergo mutations affecting growth rate, membrane permeability, capsular polysaccharide production, phage-binding receptor, and virulence. Mutations affecting the phage-binding receptor are most common and consequently prevent the phage from binding to the bacterial surface. Other identified mechanisms of phage-resistance include blocking the entry of phage genetic material or cleaving of inserted phage genetic material by restriction enzymes, the CRISPR/Cas system and other recently identified sophisticated anti-phage defense systems. In some cases, the bacterial cell can discard unassembled phage particles from the cell. This mechanism results in destroying the bacterial cell but prevents further infection by the phage. Although phage-resistance is a prominent barrier in phage therapy, the changes leading to resistance have in some cases increased susceptibility to antibiotics as well as decreased bacterial virulence [18, 19].

Jumbo bacteriophages are tailed, large-sized phages with a genome size of 200–500 kb. Most isolated jumbo phages infect gram-negative bacteria. Jumbo phages have long life cycles and they diffuse poorly in soft agar, thus producing tiny plaques that can be difficult to detect. Therefore, they may remain unnoticed in phage enrichments, and consequently the number of characterized jumbo phages is smaller than that of smaller, less than 200 kb, phages [20–22]. According to the NCBI Nucleotide Database [23], only approximately twenty *Klebsiella* jumbo phage genomes are available to date (8 Apr 2024).

When testing the sensitivity of Finnish clinical bacterial strains to commercial Russian and Georgian phage cocktails (unpublished data), one cocktail was effective against a *K. aerogenes* strain which we had no phages. According to the manufacturer, the cocktail contained no *Klebsiella* specific phages. This was interesting and prompted us to isolate and characterize the *K. aerogenes* -specific phage from the cocktail.

Here we present a novel *K. aerogenes* jumbo phage, fENko-Kae01 with a 360 kb genome. Additionally, we isolated and characterized five genetically different phage resistant bacterial mutants that allowed to identify the flagellum as the receptor to which fENko-Kae01 binds to initiate infection.

## Materials and methods

### Bacterial strains, phage cocktail and phage isolation

The bacterial strains used in this work are listed in Supplementary Table S1. All bacteria and phage incubations were performed at 37 °C using Lysogeny Broth (LB). LB agar plates included additional 1.5% agar and soft agar medium included additional 0.4% agar [24].

fENko-Kae01 was isolated from a commercial phage cocktail product (ENKO Bacteriophage, E-0116, production date: 03/2016, expiration date: 07/2017) of the Eliava Institute, Georgia, using the clinical *K. aerogenes* strain S6737 as the host (Supplementary Table S1). This *K. aerogenes* strain was subsequently used as a standard host strain for fENko-Kae01. The bacterial strain was first incubated with the phage cocktail overnight and the phage was isolated using three rounds of plaque purification [24].

The phage lysates were produced from liquid cultures. 20 µl phage suspension and 200 µl overnight incubated host bacteria were added to 5 ml LB and incubated at 37 °C with vigorous agitation for 5 h or until lysis occurred. To kill remaining bacteria, 200 µl of chloroform was added to each 3 ml of lysate, and the mixture was incubated at room temperature for 20 min by gently turning the tube up and down. The lysate was then centrifuged at 5000 rpm for 10 min or until the supernatant was clear. The supernatant was filtered through a 0.2 μm filter. To stabilize the phage during long-term storage, sucrose was added to 8%. Filtered phage lysate in LB was used for the assays unless otherwise stated.

### Electron microscopy

A purified phage lysate in 0.1 M ammonium acetate, pH 7, containing 1.8 × 10^10^ PFU/ml was prepared. LB was changed to 0.1 M ammonium acetate via ultrafiltration (Sartorius Vivaspin 300,000 MWCO ultrafiltration tubes). 3 µl of the phage preparation was transferred on to a carbon-coated copper grid and allowed to absorb for one minute. The grid was stained with 2% uranyl acetate for 30 s. The sample was then examined with a transmission electron microscope (JEOL JEM-1400, Tokyo, Japan) under 80 kV at the Electron Microscopy Unit (Institute of Biotechnology, University of Helsinki, Helsinki, Finland). Pictures were taken using Gatan Orius SC 1000B bottom-mounted Charged Coupled Device (CCD)-camera (Gatan Inc., Pleasanton, CA, USA). Ten virions with contracted and ten with non-contracted tails were measured using ImageJ (release 1.52o) [25] to determine the size and morphology of fENko-Kae01.

### DNA isolation and genome analysis

Phage DNA was isolated by phenol-chloroform extraction [24]. Briefly, 1.3 µl DNase (1 U/µl) and 4 µl RNase A (1 mg/ml) was added to 400 µl phage lysate and incubated at 37 °C for 30 min. 16 µl 0.5 M EDTA, 1.2 µl Protease K (20 mg/ml) and 20 µl 10% SDS was added and incubated at 56 °C for 60 min. After cooling to room temperature, 1 volume of phenol was added. The suspension was gently mixed by gently turning the tube up and down for 15 min at room temperature. The sample was centrifuged at 5000 rpm 5 min or until supernatant was clear. The upper phase was recovered. The extraction of the aqueous phase was repeated first with 1 volume phenol-chloroform (1:1) and then with 1 volume chloroform. Then 0.1 volume 3 M NaOAc, pH 7 and 2 volumes absolute EtOH was added to precipitate the DNA. The tube was gently turned up and down until a DNA thread was visible. The DNA thread was transferred into 1 ml 70% EtOH and centrifuged at full speed 20 min. The supernatant was removed, and the pellet was air-dried. DNA was dissolved into 100 µl TE-buffer.

Phage DNA was sequenced at Eurofins GATC Biotech. A 100,000-read subset was made out of 20,941,274 original reads with Chipster [26] at IT Center for Science, Finland (CSC) and used for assembly with A5-miseq integrated pipeline [27] for the *de novo* assembly of microbial genomes with average read coverage of 43.75. PhageTerm [28] was used to estimate the genome origin and packaging method of the phage. Sequence was verified by mapping all the original reads against the final sequence with Geneious Prime^®^ 2022.1.1 mapper. Preliminary annotation was carried out with Prokka [29]. The gene prediction annotations were double-checked with Artemis (release 17.0.1) [30]. The protein annotations were additionally checked manually with BLASTp [31] and HHpred [32]. The genome was managed with Geneious Prime (release 2023.2.1) (https://www.geneious.com). The genome was screened for tRNA (tRNAscan-SE 2.0 [33], Aragorn v1.2.38 [34]), virulence genes (VirulenceFinder 2.0 [35]) and antibiotic resistance genes (ResFinder 3.1 [36]). All the bioinformatic tools were applied using standard parameters.

The annotated genomic sequence of fENko-Kae01 was submitted to NCBI GenBank under the accession number OR228459.1.

To study the similarity of fENko-Kae01 to known phages, the whole genome was first analyzed with Microbial Nucleotide BLAST, and 30 most similar phage genomes were then selected for phylogeny analysis. Phylogeny analysis was conducted with the VICTOR Virus Classification and Tree Building Online Resource [37] using the Genome-BLAST Distance Phylogeny (GBDP) method [38] under settings recommended for prokaryotic viruses [37]. The resulting intergenomic distances were used to infer a balanced minimum evolution tree with branch support via FASTME including Subtree Pruning and Regrafting (SPR) postprocessing [39] for the formula D0. Branch support was inferred from 100 pseudo-bootstrap replicates each. Trees were rooted at the midpoint [40] and visualized with ggtree [41]. Taxon boundaries at the species, genus and family level were estimated with the OPTSIL program [42], the recommended clustering thresholds [38] and an F value (fraction of links required for cluster fusion) of 0.5 [43]. The phylogenomic tree results as well as suggestions for the classification at the species, genus and family level were all obtained directly from VICTOR and do not necessarily reflect actual ICTV taxonomy. Additionally, a heatmap integrating the intergenomic similarity values and information regarding the genome lengths and the aligned genome fraction was prepared using VIRIDIC program [44].

### Sample preparation for proteomics analysis

fENko-Kae01 did not tolerate glycerol-gradient ultracentrifugation and was therefore purified using Sartorius Vivaspin 300 000 MWCO ultrafiltration tubes. The phage lysate was washed three times with gelatin-free SM-buffer (100 mM NaCl, 10 mM MgSO_4_, 50 mM Tris-HCl, pH 7.5). The buffer was changed from LB to gelatin-free SM-buffer and the sample was concentrated so that the phage titer was 4 × 10^9^ PFU/ml.

The LC-MS/MS analysis of tryptic peptides was carried out at the Proteomics Unit, Institute of Biotechnology, University of Helsinki, Finland. The sample was reduced with 5 mM Tris(2-carboxyethyl)phosphine hydrochloride (Cat.no. 20,490, Thermo Scientific), alkylated with 10 mM iodoacetamide (Cat.no. 122,271,000, Acros Organics) in dark at room temperature, pH adjusted to pH 8.0 with 1 M NH_4_HCO_3_, and trypsin-digested at 37 °C for 16 h using Sequencing Grade Modified Trypsin (V5113, Promega). After digestion, samples were acidified with 10% trifluoroacetic acid (TFA, Cat.no. 85049.051, VWR) and desalted with BioPureSPN PROTO 300 C18 Mini columns (Cat.no. HUM S18V, Nest Group) according to manufacturer’s instructions. After desalting the samples were dried in a centrifuge concentrator (Concentrator Plus, Eppendorf). The dried peptides were reconstituted in 30 µl buffer A (0.1% (vol/vol) TFA, 1% (vol/vol) acetonitrile (Cat.no. 83640.320, VWR) in HPLC grade water (Cat.no. 10,505,904, Fisher Scientific).

For MS analysis the sample was first diluted 1:10 in buffer A1 (0.1% FA in HPLC grade water) prior to loading 20 µl to the Evotips.

### Mass spectrometry and data analysis

The desalted samples were analyzed using the Evosep One liquid chromatography system coupled to a hybrid trapped ion mobility quadrupole TOF mass spectrometer (Bruker timsTOF Pro, Bruker Daltonics) [45] via a CaptiveSpray nano-electrospray ion source (Bruker Daltonics). An 8 cm × 150 μm column with 1.5 μm C18 beads (EV1109, Evosep) was used for peptide separation with the 60 samples per day methods (21 min gradient time). Mobile phases A and B were 0.1% formic acid in water and 0.1% formic acid in acetonitrile, respectively. The MS analysis was performed in the positive-ion mode with DDA-PASEF-short_gradient_0.5s-cycletime –method [45].

For the MS data-analysis Fragpipe (version 19.1) pipeline with MSFragger (version 3.7) [46] was used. Settings in MSFragger for analysis were kept default settings except that the precursor mass tolerance was set from − 50 to 50 ppm and the fragment mass tolerance to 20 ppm. Enzyme specificity was set to “stricttrypsin” and two missed cleavages were allowed. Isotope error was set to 0/1/2. Peptide length was set from 5 to 50, and peptide mass was set from 200 to 5000 Da. The searches were done against the amino acid sequences of the predicted gene products of phage fENko-Kae01.

Based on the LC-MS/MS analysis, proteins which fulfilled the inclusion criteria (the protein probability ≥ 0.99, number tryptic peptides ≥ 2, either with the combined spectral count ≥ 4 or ≥ 2) were counted as identified proteins.

### Host range screening

A total of 156 bacterial strains, including 50 *K. aerogenes*, 53 *Enterobacter cloacae*, 10 *Echerichia coli*, 10 *Klebsiella pneumoniae*, 10 *Salmonella typhimurium* and 7 *Proteus mirabilis* strains were used to assess fENko-Kae01 host range. The host range screening was done with a liquid culture method using Bioscreen C analyser (Growth Curves AB Ltd, Finland) absorbance plate reader. The assay was performed as previously described with slight modifications [47]. The phage lysate was diluted to 1 × 10^9^ PFU/ml. Bacterial overnight cultures were diluted 1:500 in LB. 10 µl phage lysate and 190 µl diluted bacterial suspension was then pipetted into Honeycomb2-plate wells (Growth Curves AB Ltd) corresponding to MOI (multiplicity of infection) of approximately 3. A negative control containing bacteria but no phage, a positive control with *K. aerogenes* S6737 host strain and phage, and a blank control containing only LB were included to each plate. The samples were analyzed as duplicates. The results were analyzed as previously described at the 5 h timepoint [47]. The blank was subtracted from all samples and the mean was calculated from the two parallel samples. fENko-Kae01 was considered to efficiently infect a given bacterial strain if the absorbance of the culture containing phage and bacteria was < 50% of the negative control containing bacteria but no phage. The phage was considered to infect with low efficiency if the culture absorbance was 50–70% of the negative control and to not infect a given bacterial strain if the culture absorbance was > 70% of the negative control.

### Isolation of phage resistant mutants

Phage resistant bacterial mutants were isolated by incubating the *K. aerogenes* host strain overnight within a soft-agar layer containing surplus of phage fENko-Kae01 to produce a fully confluent lysis within which individual phage resistant mutants formed colonies. Morphologically differing single colonies were picked and subcultured three times to eliminate residual phage particles and to obtain pure cultures. The fENko-Kae01-resistance of the mutants was confirmed with the double layer titration method.

### Bacterial DNA extraction, sequencing, and genome analysis

Bacterial DNA was extracted from the *K. aerogenes* host strain and phage resistant mutants using the Invitrogen Jetflex Genomic purification kit and Macherey-Nagel Nucleo spin Microbial DNA kit. The DNAs of the wild type *K. aerogenes* strain (S6737) and five phage resistant mutants, S6737-M1, -M2, -M3, -M5 and -M6, were subjected to whole genome sequencing (WGS) at Novogene UK. The genomic DNA was randomly sheared into short fragments. The obtained fragments were end repaired, A-tailed and further ligated with Illumina adapter. The fragments with adapters were PCR amplified, size selected, and purified. The DNA libraries were sequenced using the Illumina paired-end 150 sequencing platform NovaSeq PE150. The trimmed and cleaned raw reads were then delivered by Novogene, and used for bioinformatics analyses. Altogether approx. 10.0, 9.43, 7.32, 7.06, 7.30, and 11.84 million reads were received for S6737, S6737-M1, S6737-M2, S6737-M3, S6737-M5, and S6737-M6, respectively.

One million sequencing reads of the wild type strain S6737 were de novo -assembled using the Geneious assembler (Geneious Prime vs. 2023.2.1) resulting in 272 contigs, 20 of which were longer than 3 Kb, with a total sequence length of 4.97 Mb. The consensus sequences of these contigs were used as reference sequences against which all the WGS reads of the phage resistant mutants were mapped using the Geneious assembler. All the SNPs, insertions and deletions against the 20 contigs were identified for each mutant. Those differences that were not common to all mutant strains were regarded as mutations occurring in genes that were the plausible causes for the phage resistance. The corresponding genes were identified from the *K. aerogenes* strain KCTC2190 genome (GenBank acc no NC_015663). The raw reads of the *K. aerogenes* host stain and of the fENko-Kae01-resistant mutants were submitted to NCBI Sequence Read Archive (SRA) under the BioProject accession number PRJNA1044302 (Supplementary Table 2).

### Phage adsorption assay

Phage adsorption assay was performed to verify whether the fENko-Kae01 resistance of the bacterial mutants was due to mutations affecting the phage-binding receptors. The host strain and phage resistant mutants were grown to OD_600_ ∼1.0, then 3.2 × 10^7^ PFU of fENko-Kae01 was added to 500 µl of bacteria, corresponding to MOI of approximately 0.1. The suspension was incubated at room temperature for 10 min and centrifuged at 13,000 g for 3 min. The supernatant was recovered and treated with 300 µl chloroform for 15 min. The sample was centrifuged as before, and the supernatant was recovered. The phage titer of unadsorbed phages was determined. The titration was performed in triplicate. LB was used as a non-adsorbing control.

### Motility assay

Motility assay was performed on the host bacterial strain and mutant strains to determine the motility of the strains. For the assay, 5 µl of bacterial culture was inoculated into LB soft agar plates (25 ml of 0.4% LB agar cast in a 9 cm diameter Petri dish). The plates were incubated at 37 °C overnight, and the diameters of the colonies were measured. The assay was performed in triplicate for each bacterial strain. The diameter of each colony was measured from three different directions. The means and standard deviations were calculated using the three measurement results of all three parallel colonies.

## Results and discussion

### Isolation and morphology

**Fig. 1:**
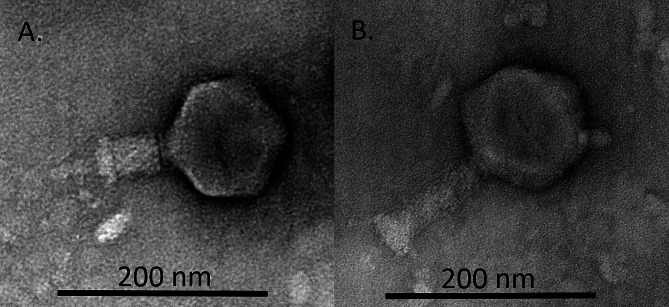
Transmission electron micrograph of fENko-Kae01. (**A**) With contracted tail. (**B**) With non-contracted tail

The phage fENko-Kae01 was isolated from a commercial phage cocktail due to its ability to infect the clinical *K. aerogenes* strain S6737 (Supplement Table S1). Electron microscopy of fENko-Kae01 revealed that the phage had an icosahedral head with a contractile tail (Fig. 1). The average total length of the non-contracted phage particle was 242.56 nm (SD 5.95) and 183.65 nm (SD 7.97) for a contracted particle without tail tube. The average length for a contacted tail was less than half of the average of a non-contracted tail length (Table 1). The tail width of a contracted tail was greater than for a non-contracted tail. The head was very symmetrical for a non-contracted particle but for a contacted particle the head was wider than it was long (Table 1). The neck connected the head to the tail and the tail ended in a baseplate. Based on these findings, fENko-Kae01 had a myovirus morphology. Additionally, the virion dimensions of fENko-Kae01 were larger than e.g., those of T4-like myoviruses on average, indicating that it is a jumbo phage [48].

**Table 1:** The dimensions of fENkO-Kae01 with contracted and non-contracted tails (all values in nm)

Tail status	Head to tail	Tail sheath length (w/o baseplate and neck)	Tail sheath width	Head length	Head width
Non-contracted	242.56 (SD 5.95)	93.44 (SD 7.64)	29.14 (SD 5.32)	118.60 (SD 4.39)	118.08 (SD 3.92)
Contracted	183.65 (SD 7.97) (w/o tail tube)	42.78 (SD 3.04)	37.02 (SD 2.71)	113.99 (SD 2.78)	119.81 (SD 2.28)

### Phage genome analysis

fENko-Kae01 had a 360,105 bp, linear, double stranded DNA genome encoding 578 predicted genes and is hence a jumbo phage. PhageTerm identified 19,736 bp direct terminal repeats that contained 50 predicted genes. The CG-content was 35.6%.

Based on bioinformatic searches, 15 of the predicted genes were identified to code virion structural proteins and 67 non-structural proteins with functions in replication, recombination, repair, translation and transcription. Thus, altogether 496 genes were annotated to encode hypothetical proteins or proteins of unknown function. tRNAscan identified 8 tRNA genes (Glu, Lys, Met, Pro, Ser, Ser, Thr, Tyr) located between bp 268,980 − 288,257.

VirulenceFinder and ResFinder did not identify any known genes encoding virulence-, toxicity-, or antibiotic resistance-associated proteins. Additionally, no genes encoding lysogeny-associated proteins were identified and therefore fENko-Kae01 can be considered to be strictly lytic and safe for phage therapy.

**Fig. 2:**
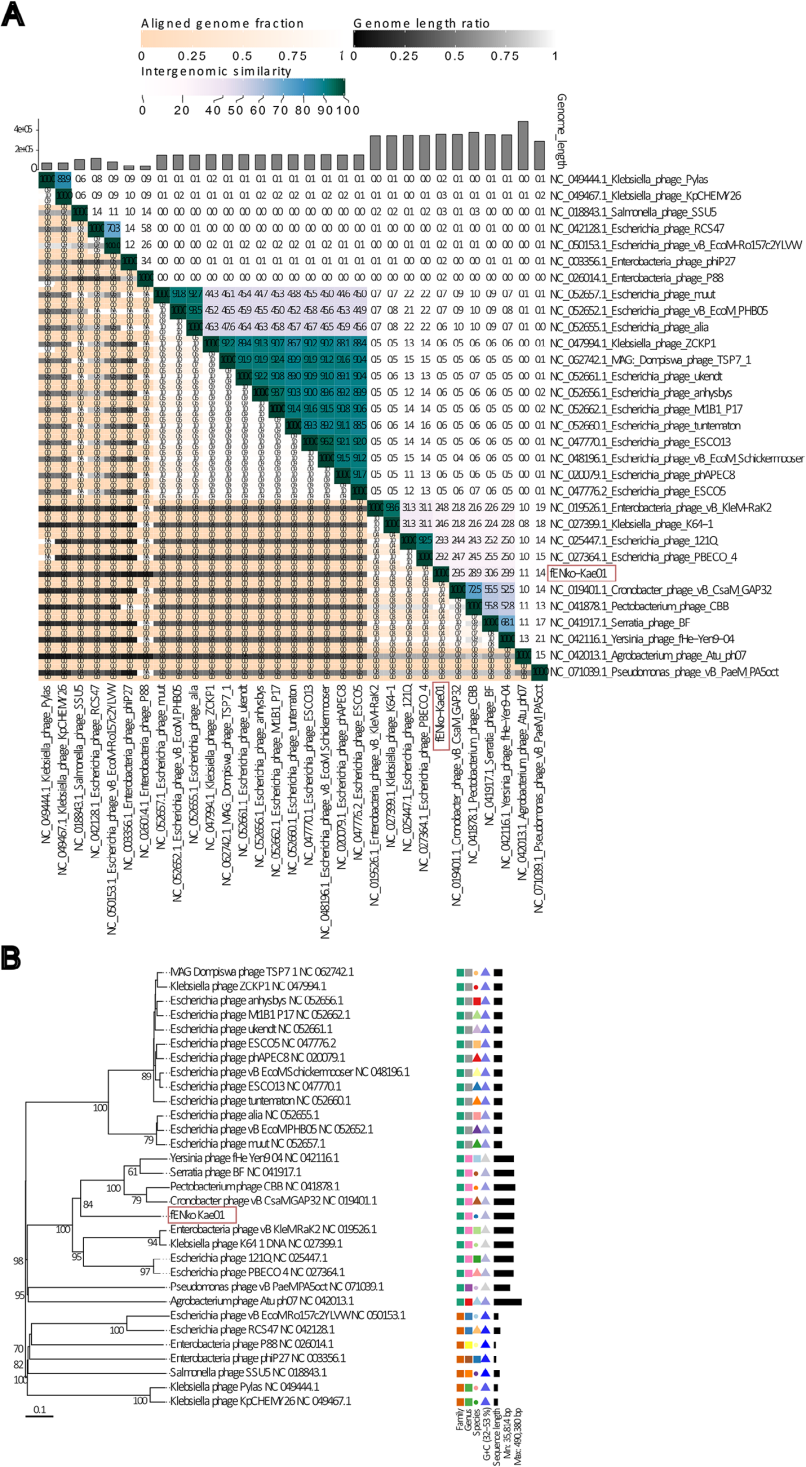
Phylogenetic tree and heat map of fENko-Kae01(red) and 30 closest related phage genomes based on BLASTn. (**A**) VIRIDIC heatmap based on intergenomic similarities between fENkO-Kae01 and closest relatives. (**B**) Phylogenetic tree using the distance formula D0, generated by VICTOR. fENko-Kae01 indicated with red box. The colors of the symbols in the “species” column indicate whether phages belong to the same or different species within the same family and genus, as proposed by VICTOR

A comparison of fENko-Kae01 genome and 30 closest phage genomes using VIRIDIC showed a sequence identity below 31% for all phages when compared to fENko-Kae01 (Fig. 2A). Phages with the highest sequence identity are also jumbo phages. A phylogeny tree generated by VICTOR using the distance formula D0 is presented in Fig. 2B. The tree shows that phages with the highest sequence identity cluster together. The four closest relatives to fENko-Kae01 according to the phylogeny tree also had the highest sequence identity to fENko-Kae01 in VIRIDIC analysis. These four jumbo phages belong to *Caudoviricetes*. No highly similar phage genomes were identified and therefore fENko-Kae01 is a novel bacteriophage. Additionally, as the sequence identity was below 31% for the closest relatives, fENko-Kae01 may represent a previously unknown genus.

Altogether, the LC-MS/MS assay identified 220 phage proteins of which 128 fulfilled the inclusion criteria of the combined spectral count ≥ 4 (Supplementary Table S3). The gene products identified by bioinformatics as structural proteins, e.g., the major capsid protein, the portal protein and 9 tail proteins were also identified as structural proteins in the proteomic analysis. Among the identified non-structural proteins were DNA polymerase, DNA topoisomerase, RNA polymerase, DNA ligase, RNA ligase, several kinases, ribosome-associated inhibitor, thymidylate synthase, a sigma factor and nucleases. Similar findings have been made with other (jumbo) phages subjected to proteomic analysis. It is speculated that these proteins are injected into the host bacteria along with the DNA to take over the host cell metabolism [49–51]. Altogether 87 proteins of unknown function were also identified.

On the other hand, when the threshold of ≥ 2 combined spectral counts was used, the total number of identified proteins increased to 158. As among the 30 additionally identified proteins were the head completion protein (CDS_236) and two baseplate wedge proteins (CDS_267 and CDS_271). This increased the number of identified proteins of unknown function to 113 (Supplementary Table S3). However, one has to keep in mind that the phage did not tolerate purification by ultracentrifugation and was purified for proteomic analysis by ultrafiltration with 300 kDa cut-off only. Therefore, the proteomic analysis shows all proteins that are part of macromolecular complexes larger than 300 kDa, and no definite conclusions whether a given protein is a part of the phage particle can be made.

### fENkO-Kae01 host range

Phages are host specific and often only infect one bacterial species and only certain strains of this bacterial species. Of the *K. aerogenes* strains used in the host range assay, fENko-Kae01 infected efficiently only 2 strains (4%) and with low efficiency another 6 strains (12%) (Supplementary Table S1). These 8 strains were all human blood culture isolates. fENkO-Kae01 did not infect any of the other bacterial species used in the assay. fENko-Kae01 seems to have a relatively narrow host-range which can limit its applicability in phage therapy. However, host-range expansion protocols have been developed to overcome the limitations of narrow host-range phages [52, 53]. As the phage was isolated from a commercial phage cocktail that was not targeting *K. aerogenes*, it is likely that fENko-Kae01 does in fact infect other bacterial species with a suitable phage binding receptor. Interestingly, the long tail fiber protein of fENko-Kae01 (WNV47359) was approx. 99% identical to respective proteins of *Salmonella* phages 7t3 and Munch (protein accession numbers QCW18942 and EAZ2023036, respectively), but the phage did not infect any *Salmonella* strain in our collection. We only identified one gene encoding long tail fiber in fENko-Kae01 genome, which is in contradiction to some other jumbo phages and probably explains the narrow host range. For example, *Klebsiella aerogenes* -specific jumbo phages 4Kp5130 and 4Kp9438 and *Dickeya solani* -specific jumbo phages JA11, JA13, and JA29 have several putative tail fiber proteins, which enables them to infect a wide range of strains even spanning capsular type or species borders [54, 55].

### Phage resistant host mutants

Phage resistant mutants emerged when the host strain was incubated in the presence of fENko-Kae01. Five different phage resistant mutants (S6737-M1, -M2, -M3, -M5 and -M6) were isolated and subjected to WGS. Genomic changes that differed from those of the original host strain S6737 could be identified from all the mutants (Table 2). Specifically, in mutants S6737-M1, -M2, -M5 and -M6, the mutations had affected genes encoding proteins with different functions in the flagellum biosynthesis, and the mutant S6737-M3 had a deletion inactivating the Rcs regulatory system genes *rcsB* and *rcsC*.

**Table 2:** Mutations leading to fENko-Kae01 resistance

Strain	S6737-M1	S6737-M2	S6737-M3	S6737-M5	S6737-M6
Nature of the mutation	A ca. 600 bp deletion within the *fliC* gene	A 71 bp deletion at the *flgG-flgH* intergenic region, inactivates the *flgH* gene	*rcsB-rcsC* 800 bp deletion	*fliG* I121S substitution	A substitution in the *flhA* gene that generates a premature stop codon

**Fig. 3:**
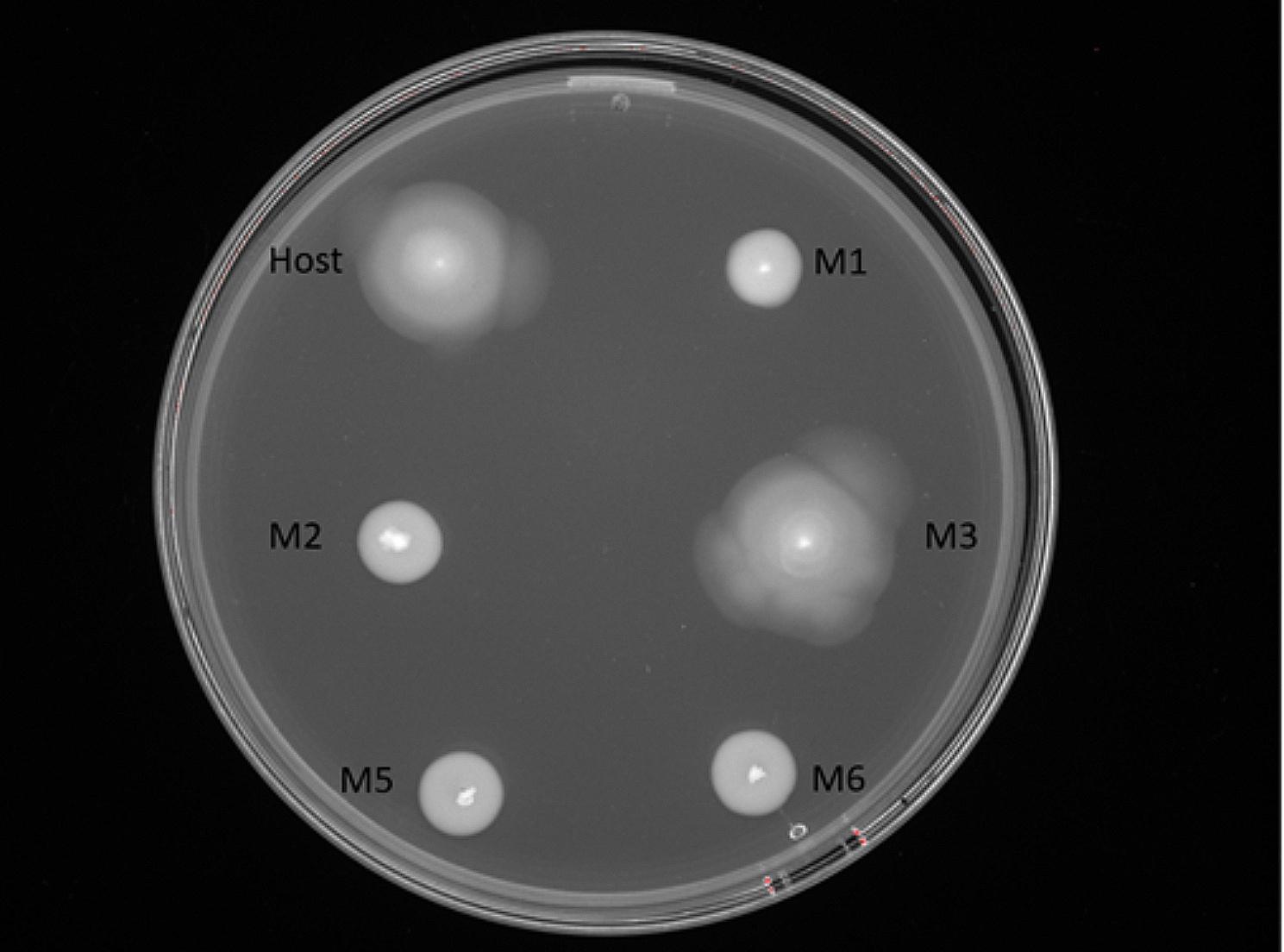
Motility of host strain (S6737) and mutant strains S6737-M1, S6737-M2, S6737-M3, S6737-M5, and S6737-M6 after overnight culture on LB soft-agar (0.4% agar)

A motility assay was performed on the host bacterial strain and mutant strains to determine whether the strains were motile or non-motile. The colonies of the strains cultured overnight on soft-agar plates had following diameters (mm): S6737 29.61 (SD 4.23), S6737-M1 12.83 (SD 1.03), S6737-M2 7.89 (SD 0.96), S6737-M3 30.11 (SD 6.49), S6737-M5 8.06 (SD 0.63) and S6737-M6 7.50 (SD 0.35). The results demonstrated that the host strain S6737 and the Rcs-mutant strain S6737-M3 were fully motile while the four strains with mutations affecting the flagellar proteins were non-motile (Fig. 3). This confirmed the results obtained from the genome analysis indicating that lack of any of the proteins FliC (flagellin, structural protein of the flagellar filament), FliG (major component of the cytoplasmic C ring), FlhA (flagellar secretion apparatus component), and FlgH (component of the L ring) prevented the expression of functional flagellum. Interestingly, while other genes were inactivated by distinct deletions that prevent the production of the proteins, the mutation in S6737-M5 was a single nucleotide substitution in the *fliG* gene causing a I121S substitution. As the presence of complete flagellar biosynthesis operons were verified for each isolate during the genomic analysis, this superficially minor substitution in the *fliG* gene must influence the intricate protein-protein interactions in the flagellar function.

**Fig. 4:**
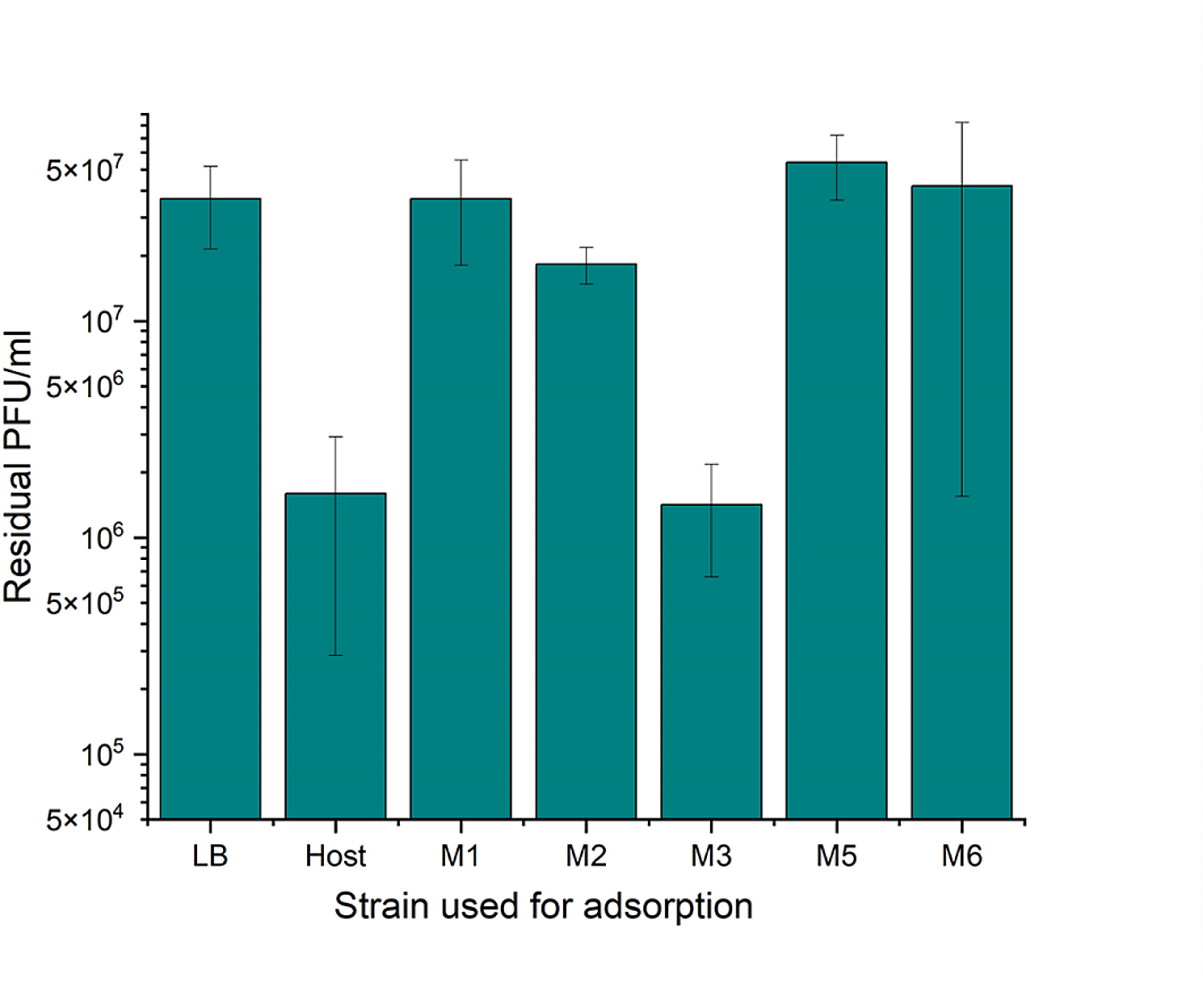
Adsorption of fENko-Kae01 to original host and phage resistant strains. Shown are the numbers of non-adsorbed fENko-Kae01 phages in the supernatants after incubation with the host strain S6737 and the phage resistant mutants S6737-M1, S6737-M2, S6737-M3, S6737-M5 and S6737-M6. LB indicates an adsorption-free control without bacteria. Columns indicate mean residual PFU/ml of three parallel titrations and error bars indicate SD. Calculations and visualization were performed with OriginPro 2023b (OriginLab Corporation)

Phage adsorption assays were performed to verify whether the phage fENko-Kae01 resistance mutations affected the phage receptors (Fig. 4). While there was practically no adsorption of phage fENko-Kae01 to the non-motile mutant stains S6737-M1, -M2, -M5 and -M6, the phage adsorbed at the wild type level to mutant S6737-M3. Thus, the *rcsBC-*mutation, causing phage resistance in this strain, did not affect the expression of the phage receptor.

Based on the sequence analysis and motility- and adsorption assay results, fENko-Kae01 seemingly uses the bacterial flagellum as its phage receptor. The putative other receptor(s) of fENko-Kae01 on the bacterial cell surface remain unidentified. The majority of mutations that result in phage resistance are receptor mutations that inhibit the phage from binding to the receptor and hence infection [18]. This was also observed for the isolated phage resistant strains in this study.

The Rcs regulatory system is critical for gene expression in bacteria. The RcsB and RcsC proteins in Enterobacteriaceae species have been identified to have a role in capsule production and biofilm formation [56]. The RcsB and RcsC proteins also negatively regulate the *flhlDC* operon, that in turn positively regulates many of the genes for flagellar synthesis [56]. Mutations affecting the RcsB protein have been shown to increase bacterial motility [57, 58]. In the motility assay performed in this study we can see a slightly increased motility in mutant S6737-M3 compared to the wild type which is consistent with earlier findings. The Rcs is a complex phosphorelay regulatory system that to date is not yet that well known and hence it is possible that the mutation affecting the Rcs system could in some other way inhibit the phage from injecting its DNA into the cell or inhibit the production of phage particles.

## Conclusions

A novel lytic, *K. aerogenes* specific jumbo phage, fENKo-Kae01, was isolated from a commercial phage cocktail. fENko-Kae01 seems to be a completely new genus representative. The phage uses the bacterial flagellae as its phage-binding receptor. fENko-Kae01 is considered safe for phage therapy but its relatively narrow host range, the emergence of phage resistant bacterial mutants when bacteria were cultured with high phage concentrations, and the instability of the phage in ultracentrifugation may limit its therapeutical potential and the available purification methods that can be applied to the phage. This information is valuable if the phage is used for phage therapy.

### Electronic supplementary material

Below is the link to the electronic supplementary material.

Supplementary Material 1

## Data Availability

All data presented in the study are included in this published article and its supplementary information files. The genomic sequence of phage fENko-Kae01 has been submitted to the NCBI Gen Bank database under the accession number OR228459.1. Raw sequencing reads of host bacterial strain and phage resistant bacterial mutants have been submitted to the NCBI Sequence Read Archive (SRA) under the BioProject accession number PRJNA1044302. Further inquiries can be directed to the corresponding author.
